# 肺癌的免疫治疗临床研究进展

**DOI:** 10.3779/j.issn.1009-3419.2012.10.08

**Published:** 2012-10-20

**Authors:** 莎 金, 建辉 田

**Affiliations:** 1 201203 上海，上海中医药大学2011级研究生 Graduate Student of Shanghai University of Traditional Chinese Medicine, Shanghai 201203, China; 2 200032 上海，上海中医药大学附属龙华医院肿瘤科 Department of Oncology, Longhua Hospital Affiliated to Shanghai University of Traditional Chinese Medicine, Shanghai 200032, China

**Keywords:** 肺肿瘤, 主动免疫治疗, 被动免疫, 免疫逃逸, Lung neoplasms, Active immunotherapy, Passive immunization, Immune escape

## Abstract

肺癌是一种恶性程度很高、早期发现率低且预后很差的恶性肿瘤，迫切需要开发更有效的治疗方法和药物。随着对肿瘤免疫发生和免疫耐受认识的不断深入，肺癌的免疫治疗进展很快，早期临床研究初步确认了依普利单抗、程序性死亡受体抗体（programmed death-1, PD-1）、Toll样受体拮抗剂、BLP25脂质体疫苗、Belagenpumatucel-L、黑色素瘤相关抗原A3（melanoma-associated antigen A3, MAGE-A3）蛋白疫苗和乳铁蛋白等对肺癌的治疗有效性，多数已进入Ⅲ期临床研究阶段，有望获得更有力的证据而最终成为肺癌综合治疗的重要组成部分。肺癌免疫治疗疗效提高面临的问题有目标人群的选择、免疫耐受的克服（包括免疫逃逸和免疫衰老）、免疫治疗疗效标准的制订等。

## Abstract (en)

Lung cancer is a highly malignant disease with poor prognosis, most cases are diagnosed at a very late stage. More effective medications or therapies should be developed to improve its prognosis. The advancement of tumor immunity and tumor immunosuppression facilitated the feasibility of immunotherapies for lung cancer. Ipilimumab, antibody to Programmed death-1 (PD-1), Toll-like receptor agonists, liposomal BLP25 (L- BLP25), belagenpumatucel-L, melanoma-associated antigen A3 (MAGE-A3) vaccine and talactoferrin have been proved to be effective for lung cancer through early clinical trials, most of the drugs have moved forward to Phase Ⅲ trials, so as to collect much higher level evidence to support the immunotherapies incorporated into the multidisciplinary treatment of lung cancer. The selection of target patients at appropriate stages, breaking down of tumor immunosuppression as well as the objective measurement of tumor response to the therapy are major challenges for the development of immunotherapies for lung cancer. The clarifying of the mechanism of immune escape led to the above drug development, and immune-senescence has already become the hotspot in this field.

最新肿瘤流行病学调查^[1]^显示，男性原发性支气管肺癌的发病率和死亡率居所有恶性肿瘤首位，女性肺癌死亡率也高居所有恶性肿瘤第二位。由于肺癌的恶性程度较高，并且60%的患者在确诊时已属中晚期，失去手术机会而预后极差，发展中国家肺癌患者的5年生存率仅8.9%，手术、化疗和放疗为主的综合治疗的疗效并不令人满意，寻找肺癌治疗的新策略成为研究的焦点^[2]^。随着分子生物学和免疫学理论及科研技术的发展，免疫治疗再次引起了研究者浓厚的兴趣。2008年《新英格兰医学杂志》报道1例晚期黑色素瘤的免疫治疗取得成功^[3]^，该患者为全身多处转移病例，经自身CD4^+^T细胞治疗后所有病灶消失，经随访26个月长期生存。2011年的诺贝尔医学奖也授予从事肿瘤免疫治疗相关的3位科学家，预示了免疫治疗在恶性肿瘤治疗领域的广阔前景。但时至今日，美国食品药品监督管理局和欧洲药物学会尚未批准针对肺癌的疫苗或者免疫治疗技术，提示肺癌的免疫治疗仍需要大量深入的研究。

## 1 肺癌免疫治疗有效性得到初步确认

长期以来免疫治疗对肺癌的作用存在争议，学者们认为肺癌是一种免疫原性较弱的恶性肿瘤^[4]^。研究者^[5]^对188例肺癌标本进行的DNA测序发现，26个常见的突变基因均与免疫无关，故而认为免疫治疗用于肺癌缺乏生物学基础难以取得理想的效果。但是这种观点随着免疫学的基础和临床研究而受到了挑战，并且由于免疫治疗具有高度特异性和维持患者长期生存的潜在优势，正吸引了不少学者潜心于该领域的研究^[6]^。

最新的一项荟萃分析^[7]^评估了免疫治疗对进展期非小细胞肺癌（non-small cell lung cancer, NSCLC）的疗效，取得了令人鼓舞的结果。研究包括12项随机临床试验共计3, 134例肺癌患者，其中有1, 570例男性和1, 564例女性患者，均经病理学证实为Ⅲa期、Ⅲb期和Ⅳ期的进展期肺癌患者。研究者将总生存期（overrall survival, OS）、无疾病进展生存期（progression free survival, PFS）、完全缓解、部分缓解和总有效率确定为有效终点指标。结果发现与对照组比较，免疫治疗组（单克隆抗体、细胞因子和疫苗）的未分层OS、PFS、部分缓解率和总有效率明显改善（*P*分别为0.000, 7，0.000, 4，0.002，0.003），但完全缓解率改善并不明显（*P*=0.97）。亚组分析发现单克隆抗体可明显改善患者的PFS、部分缓解率和总有效率，并且显示出改善OS的趋势。研究仅观察到1项明显的免疫治疗相关不良反应。未发现疫苗与对照组在不良反应方面的差异，而细胞因子疗法可诱发3种严重不良反应。从而认为免疫治疗对进展期NSCLC有明确疗效，如能解决过敏和不良反应问题，单克隆抗体有望成为肺癌的标准补充治疗，丰富肺癌综合治疗的内容。

## 2 肺癌的单克隆抗体治疗

### 2.1 依普利单抗

依普利单抗Ipilimumab是针对细胞毒T淋巴细胞相关抗原4（cytotoxic T-lymphocyte-associated antigen 4, CTLA-4）的一种单克隆抗体。正常情况下T细胞激活后可表达CTL-4，后者与同样表达T细胞表面的CD28分子竞争性结合位于抗原呈递细胞（antigen presenting cells, APC）表面的B7家族免疫分子，从而抑制B7家族分子与CD28的结合效率，进而影响T细胞的活化，最终降低细胞毒性T细胞的肿瘤杀伤效力。依普利单抗可通过阻断CTLA-4与其配体B7分子的结合，从而可以促进T淋巴细胞的活化与增殖，达到提高机体对肿瘤的细胞免疫和体液免疫反应的抗瘤作用^[8]^。值得一提的是，该药是近30年来首个被证明能延长晚期黑色素瘤患者生存的免疫类药物。

最近报道的一项Ⅱ期临床研究^[9]^评价了该药联合化疗治疗肺癌的活性和安全性。研究将首次接受化疗的204例肺癌患者随机分为3组：紫杉醇加卡铂联合安慰剂对照组、紫杉醇加卡铂联合同步依普利单抗治疗组（4个疗程的化疗同步使用依普利单抗，后续2个疗程的安慰剂联合化疗）、紫杉醇加卡铂联合序贯依普利单抗治疗组（2个疗程的安慰剂联合化疗后续4个疗程的依普利单抗联合化疗）。依普利单抗采用每3周1次的静脉给药方式。合格的入选患者继续进行安慰剂和依普利单抗的维持治疗。对治疗反应的判断采用免疫治疗相关反应标准和改良的世界卫生组织实体瘤疗效标准。主要终点指标为免疫相关无疾病进展生存期（immune-related progression free survival, irPFS），其它终点指标为PFS、最佳总有效率（best overall response rate, BORR）、免疫相关最佳总有效率（immune-related BORR, irBORR）和安全性评价。结果发现序贯免疫治疗的irPFS明显优于安慰剂对照组（HR=0.72, *P*=0.05），并能改善PFS（HR=0.69, *P*=0.02）。但同步免疫治疗组没有得到阳性结果（HR=0.81, *P*=0.13）。序贯免疫治疗、同步免疫治疗和对照组的中位irPFS分别为5.7个月、5.5个月和4.6个月，中位PFS分别为5.1个月、4.1个月和4.2个月，irBORR分别为32%、21%和18%，BORR分别为32%、21%和14%，中位OS分别为12.2个月、9.7个月和8.3个月，3级到4级免疫相关不良反应总发生率分别为15%、20%和6%。研究结果发现依普利单抗联合化疗可有效提高irPFS和PFS，支持进一步开展后续研究全面评价其疗效。

### 2.2 程序性死亡分子1抗体（programmed death-1, PD-1）

PD-1属于抑制性共刺激分子，表达在活化的T细胞，其受体表达在肿瘤细胞以及肿瘤微环境的基质细胞，两者结合后可诱发T细胞功能的抑制而诱导免疫逃逸。PD-1抗体阻断PD-1与其受体的结合，从而避免T细胞的免疫耐受。最近的一项研究评价了PD-1抗体对多种实体瘤的活性。该项Ⅱ期临床研究^[10]^共纳入包括晚期黑色素瘤、肺癌、肾癌在内的实体瘤共296例，给予的剂量区间为0.1 mg/kg-10 mg/kg，每2周给药1次，每8周进行1次疗效评价，纳入病例可用药至12周以上，直到出现疾病进展或者完全缓解。结果发现14%的患者出现3级到4级不良反应，没有确定最大耐受剂量。其中共236例患者可进行客观疗效评价，NSCLC的缓解率为18%，并且疗效持久。此外，在用药前检测了肿瘤标本的PD-1表达状态检测，其中17例无表达的肺癌患者无效，充分证明了该药的高度选择性。目前该药治疗肺癌的注册临床研究即将开始。

## 3 肿瘤疫苗

肿瘤细胞疫苗是以肿瘤细胞为免疫原的免疫治疗方法，一般由一个或多个肿瘤抗原成分和免疫佐剂组成。肿瘤抗原可为重组蛋白、多肽、肿瘤溶解物或者经过放射处理的肿瘤细胞。其优点是可产生较持久的免疫反应，适用于失去手术机会患者的姑息治疗或术后患者预防肿瘤复发转移治疗。

### 3.1 BLP25脂质体疫苗（L-BLP25）

L-BLP25是一种人工合成的多肽疫苗，其含有的20个氨基酸多肽与表达于肺癌细胞的粘蛋白结合后可刺激机体产生强大的免疫反应。Butts等^[11]^报道了L-BLP25疫苗治疗晚期NSCLC患者Ⅱ期随机多中心临床试验的更新生存分析结果。治疗组为皮下注射L-BLP25 930 μg，每日1次，连续8周，继以每6周1次的维持量，同时采用最佳支持治疗；对照组为单纯最佳支持治疗。结果提示治疗组中位生存期较对照组长4.2个月（*P* < 0.01），中位生存期提高到17.2个月，3年生存率为31%，而对照组中位生存期为13.0个月，3年生存率仅为17%。分层分析发现，治疗组的Ⅲb期患者中位生存期比对照组长17.3个月（30.6个月*vs* 13.3个月）。基于Ⅱ期临床研究令人鼓舞的结果，已经开始了该疫苗的Ⅲ期临床研究（NCT01015443）和专门针对亚洲肺癌患者的研究（NCT00409188），预计共累计入组患者约1, 800例^[12]^。

### 3.2 Belagenpumatucel-L

肿瘤转化生长因子-β2（transforming growth factor-β2, TGF-β2）是一种抑制因子，可抑制自然杀伤细胞（natural killer cell, NK）、活化杀伤性T细胞和树突状细胞（dendritic cells, DC）的活性，其表达水平越高则肺癌预后越差。Belagenpumatucel-L是一种转化生长因子β2反义基因修饰的同种异体肿瘤细胞疫苗。它由4种转染了TGF-β2反义基因的NSCLC细胞系构成。这种经基因修饰的肿瘤细胞系可以通过分泌TGF-β2的反义寡核苷酸从而达到增强疫苗免疫原性的目的。Nemunaitis等^[13]^报道了该药治疗NSCLC的Ⅱ期临床研究结果，证明该药具有可以耐受的毒性和明确的剂量依赖性生存受益。在2012年的美国癌症协会年会上，研究者^[14]^报道该药治疗的所有75例晚期肺癌患者的中位生存期为14.5个月，5年存活率为20%。经过一线化疗后稳定的晚期肺癌患者中位生存期为44.4个月；5年生存率达50%。由于目前接受一线治疗的肺癌患者的中位生存期为14.1个月及5年生存率为9.1%，这是目前为止最令人振奋的免疫治疗研究结果。目前正在进行该药Ⅲ期临床研究以判断其在晚期肺癌一线化疗会后维持治疗的价值，预计研究将纳入700例晚期肺癌患者（NCT00676507）。

### 3.3 黑色素瘤相关抗原A3（MAGE-A3）

蛋白疫苗MAGE-A3在包括肺癌的多种肿瘤细胞表达而在正常细胞无表达，目前被认为是真正的肿瘤特异性抗原。采用MAGE-A3重组蛋白和免疫佐剂组成的蛋白疫苗已经被用于肺癌的治疗。近期的一项Ⅱ期临床研究^[15]^共纳入182例（122例Ⅰb期和60例Ⅱ期）手术后MAGE-A3表达阳性患者，免疫治疗每3周1次，连续5次，后续每3月1次，共8次治疗。主要研究终点是无疾病间期（disease free intervals, DFI），其它终点指标是安全性、无病生存期（disease free survival, DFS）、OS。中位随访时间为28个月，共发现67例复发病例和45例死亡病例。组间比较发现MAGE-A3组的DFI、DFS和OS有优于对照组的趋势，但无统计学差异，药物毒性可以耐受。为进一步判断该药作为肺癌术后预防复发的价值，目前已经开展Ⅲ期研究（NCT00480025）^[16]^，研究将纳入Ib期、Ⅱ期和Ⅲa期术后患者，预计将从全球招募共2, 270例患者，因此会成为最大的肺癌辅助治疗临床研究项目。主要目的是判断免疫治疗预防肺癌术后复发的作用，其次要观察该疗法的疗效预测因素，该研究在我国也招募患者。

## 4 过继免疫治疗

过继性细胞免疫治疗是指向肿瘤患者输注具有抗肿瘤活性的免疫细胞，通过直接杀伤肿瘤或激发机体免疫反应来杀伤肿瘤细胞，最终达到治疗肿瘤的目的。该方法所用细胞主要包括淋巴因子激活的杀伤细胞、肿瘤浸润性淋巴细胞、CD3单克隆抗体激活的杀伤细胞、细胞因子诱导的杀伤细胞等。目前只有细胞因子诱导的杀伤细胞（cytokine induced killer, CIK）在临床研究方面较为成熟，国内已经有多家医院开展此项疗法。CIK是经体外细胞因子刺激后大量扩增的活化T细胞，具有体外大量扩增、抗瘤活性强、没有主要组织相容性抗原限制性等特点。

Li等^[17]^报道自体CIK细胞治疗肺癌的一项Ⅱ期配对临床研究，研究共纳入87对肺癌患者，其中包括50对Ⅰ期-Ⅲa期的早期患者和37对Ⅲb期-Ⅳ期的晚期患者，分为对照组（单纯化疗）和治疗组（化疗加CIK细胞疗法）。化疗采用吉西他滨或长春瑞滨或多西紫杉醇加铂类，治疗组为在每周期化疗的第15、16天采用自身CIK细胞疗法，每次静脉输入（13.07±1.37）×10^9^自身CIK细胞，每月1次治疗。2周期后评价疗效，两组均实施4个周期的维持治疗直到疾病进展。结果发现CIK疗法联合化疗没有改善早期肺癌患者的3年无疾病进展生存期（*P*=0.259），但可改善3年总生存率（82% *vs* 66%, *P*=0.049）及中位OS（73个月*vs* 53个月，*P*=0.006）。晚期肺癌患者中CIK疗法加化疗可明显延长3年PFS（13个月*vs* 6个月，*P*=0.001）与OS（24个月*vs* 10个月，*P* < 0.001）。多元回归显示CIK细胞疗法的应用频率与PFS（HR=0.91, *P*=0.012）和OS的延长密切相关（HR=0.83, *P*=0.001）。

徐永茂等^[18]^报道一项随机对照临床实验研究，78例中晚期NSCLC患者随机分成研究组38例（化疗+过继免疫治疗）与对照组40例（化疗），研究组采用长春瑞滨联合顺铂（NP）方案联合CIK细胞和DC治疗，对照组采用长春瑞滨联合顺铂（NP）方案化疗，观察两组临床疗效、毒副作用、患者生存质量、免疫功能。结果研究组有效率明显高于对照组（65.7% *vs* 40.0%, *P* < 0.05），研究组中位PFS为5.3个月，中位OS为11.9个月；对照组中位PFS为4.6个月、中位OS为10.4个月，两组无明显差异（*P*=0.12, *P*=0.15）。研究组患者的生存质量及免疫功能明显高于对照组（*P* < 0.005），而两组毒副作用无明显差别。

## 5 其它免疫疗法

### 5.1 乳铁蛋白

乳铁蛋白与肠上皮结合后被转运到淋巴组织，可募集并诱导循环系统中携带肿瘤抗原的幼稚树突状细胞早日成熟并活化，进而诱导强烈的抗肿瘤免疫反应。最近的一项Ⅰ期临床研究^[19]^纳入了100例一线化疗失败的晚期肺癌患者，治疗组口服1.5 g乳铁蛋白，每日2次，连续用药12周，休息2周为1周期，最大剂量为3个周期。对照组为安慰剂组，两组均结合常规支持疗法。主要研究终点为OS、次要研究终点为PFS和疾病控制率以及安全性。意向性分析观察到该药干预后患者的OS延长，中位总生存期延长了65%（3.7个月到6.1个月；*P*=0.04），PFS和疾病控制率也有改善的趋势。3级以上不良反应很低，且耐受性良好。旨在深入研究该药疗效的Ⅲ期临床研究已经开始，预计纳入将近720例患者（NCT00707304）。

### 5.2 肿瘤坏死因子（Tumor Necrosis Factor, TNF）

TNF-α是由激活的单核-巨噬细胞产生的一种可溶性多功能细胞因子，对多种肿瘤细胞有直接的细胞毒作用，并具有调节细胞免疫功能的作用。任莉等^[20]^观察了重组改构人肿瘤坏死因子对晚期肺癌的作用，将132例中晚期NSCLC患者随机分入试验组与对照组，两组均采用顺铂、表柔比星联合环磷酰胺（CAP）或紫杉醇联合顺铂（TP）或长春瑞滨联合顺铂（NP）方案化疗，21天为1周期，连用2个周期。在试验组化疗的第1-7天和第11-17天肌肉注射rmhTNF 500万单位。结果发现试验组与对照组有效率分别为34.6%和27.8%（*P*=0.041），中位OS分别为8.2个月和6.7个月（*P*=0.034），中位疾病进展时间6.4个月和5.9个月（*P*=0.138），1年生存率分别为51.8%和42.6%（*P*=0.039），2年生存率分别为22.6%和20.8%（*P*=0.734），3年生存率分别为15.2%和14.7%（*P*=0.863）。研究认为rmh-TNF联合化疗药物治疗NSCLC的近期疗效优于单纯化疗，中位OS和1年生存率更高，但在改善生活质量、中位疾病进展时间、2年和3年生存率方面未见明显优势。

## 6 肺癌对免疫治疗耐受的主要原因及相应的对策

虽然目前免疫治疗迅速成为肺癌临床研究的热点，但是由于以下原因，导致机体对免疫治疗产生耐受而疗效欠佳。因此，克服免疫耐受则是提高肺癌免疫治疗效果的重要策略。

### 6.1 肺癌导致免疫逃逸

肺癌细胞可通过多种途径逃避机体免疫监视，包括肿瘤抗原的丢失、HLA分子的下调，和分泌多种免疫抑制因子等。

#### 6.1.1 肿瘤细胞特异抗原表达下降及变异造成肿瘤免疫原性减弱

由于肿瘤相关抗原往往在肺癌细胞和正常细胞同时表达，导致机体免疫系统不能对携带自身抗原的肿瘤细胞的有效识别而不能产生有效的免疫反应。在肺癌细胞的进化过程中，可出现Ⅰ类人类白细胞抗原（leukocyte differentiation antigen, HLA-I）的表达降低或者缺失，导致其不能被树突状细胞有效识别^[21]^。研究发现主要组织相容性抗原（major histocompatibility complex, MHC-1）表达产物HLA-I的下降程度与肿瘤恶性程度、转移风险及预后险恶呈正相关。MHC-I表达的变化影响着肿瘤免疫治疗的疗效。对免疫治疗敏感的肿瘤患者来说，经过肿瘤免疫治疗之后肿瘤细胞MHC-I表达较治疗前有所增加；对肿瘤免疫治疗耐受的患者来说，肿瘤细胞的MHC-I表达依然很低^[22]^。前述肿瘤细胞疫苗即是由各种肿瘤抗原成分和免疫佐剂组成，其目的即是增强肿瘤细胞的免疫原性。各种免疫佐剂可能在未来的肿瘤疫苗设计中具有重要地位，如能结合药物缓释和控释技术将进一步增强肿瘤抗原的释放效率，最终达到高效、持久激活免疫反应的目的。

#### 6.1.2 肺癌细胞分泌免疫抑制因子，促进宿主免疫系统对肿瘤产生免疫耐受

肺癌细胞可主动分泌免疫抑制因子如转化生长因子TGF-β、前列腺素E2、白介素10（Interleukin-10, IL-10）和环氧和酶2、吲哚胺-2, 3-双加氧酶（Indoleamine 2, 3-dioxy- genase, IDO）、Toll样受体^[23]^等免疫抑制因子，这些因子可促进1类和2类T淋巴辅助细胞的（T helper 1, Th1/Th2）的平衡向Th2漂移，影响DC细胞的抗原呈递和表达、下调T细胞粘附和共刺激分子的表达，降低效应T细胞的活化和功能，诱导免疫耐受的发生^[24]^。一旦明确其主要信号通路，则予以阻断可逆转免疫逃逸的发生。例如IDO在肺癌微环境的高表达一方面导致T细胞停滞于生长周期的G_1_期而生长停滞，另一方面促进色氨酸下游代谢产物(如犬尿素酸)水平升高，后者可直接溶解成熟T细胞或诱导其凋亡。而沉默肿瘤细胞IDO基因后可恢复T细胞特异性抗肿瘤免疫应答能力^[25]^。目前针对IDO信号通路的拮抗剂1-MT已经证实可逆转IDO诱导的免疫逃逸，为相关新药开发提供了证据。而临床研究^[26]^发现Toll样受体9（toll-like receptor 9, TLR9）拮抗剂可给肺癌患者带来明显的生存获益。

### 6.2 免疫衰老所致的肺癌免疫治疗耐受

人体衰老的一项重要标志是免疫系统功能的不断下降，从而大大提高了对感染性疾病和肿瘤的易感性。临床研究发现肺癌的发病随着年龄增长而提高也支持这种观点^[27]^。最新研究^[28]^发现调节性T细胞（T regulatory cell, Treg）在诱导免疫衰老方面有重要作用，Treg是T细胞的一个亚型，它可通过影响效应T细胞和树突状细胞的功能而控制机体T细胞的反应，而衰老Treg可抑制效应CD4细胞的功能，降低Th1细胞因子如IL-2和IFN的表达，导致免疫衰老的发生。最近研究发现从老年小鼠的骨髓、血液和淋巴器官中提取到一种发育不成熟的髓源细胞，其表型和从进展性肿瘤中提取的髓源抑制细胞相似，这类细胞的特点是其表面同时表达Gr1和CD11b分子（Gr1^+^CD11b^+^细胞）。它可通过一氧化氮依赖通路抑制T细胞的成熟和活化，并且诱导高水平的炎性因子表达。但目前尚无临床研究资料支持。将来有望通过减少体内调节性T细胞或者Gr1^+^CD11b^+^细胞的量或者影响其功能而达到阻断免疫衰老的作用，进而提高免疫治疗的效果^[29]^。

## 7 肺癌免疫治疗未来展望

肺癌免疫治疗进展很快，很多治疗项目已经临床开展，无论从主动免疫或过继免疫角度，均有一定的研究进行，部分研究显示出良好的疗效，预示了免疫治疗在肺癌综合治疗中诱人的前景。

由于肺癌免疫治疗的机理尚待完全阐明，因此免疫治疗的剂量及疗程尚无统一标准、疗效尚待进一步证实，因此尚未纳入肺癌综合治疗的临床指南中。免疫治疗要成为肺癌的常规治疗方法尚需时日。现有的研究规模普遍比较小，多处于Ⅰ期-Ⅱ期的临床研究阶段，但可喜的是已有部分药物进入Ⅲ期临床试验阶段进一步证实免疫治疗疗效和安全性，最终有望提供更确切的证据支持免疫治疗成为肺癌综合治疗方案的组成部分。

由于免疫治疗和化学治疗的机理不同，其作用机理和特点也不同，但目前研究多数采用现行的化疗药物评价标准进行，不能客观反应免疫治疗的疗效。因此，需要在进一步明确免疫治疗原理的基础上，不断完善免疫治疗的疗效评价标准。目前有学者基于一些免疫治疗表现出的特点正在建立一种免疫治疗相关疗效评价标准，主要有以下特点：与基线数据相比肿瘤负荷减小，没有新病灶出现，疾病长期稳定和出现新病灶但对原病灶有抑瘤作用^[30]^。

目前，恶性肿瘤的综合治疗理念已经深入人心。应该广泛开展免疫治疗在预防术后复发转移、与放化疗联合应用增加效果、在肺癌维持治疗中的作用，进一步明确其适应症和不良反应。为临床推广提供详实的基础，从而丰富肺癌综合治疗的内容，最终提高其疗效。

## References

[1] Jemal A, Bray F, Center MM (2011). Global cancer statistics. CA Cancer J Clin.

[2] Ramalingam S, Belani C (2008). Systemic chemotherapy for advanced non-small cell lung cancer: recent advances and future directions. Oncologist.

[3] Hunder NN, Wallen H, Cao J (2008). Treatment of metastatic melanoma with autologous CD4+ T cells against NY-ESO-1. N Engl J Med.

[4] Tomasini P, Khobta N, Greillier L (2012). Ipilimumab: its potential in non-small cell lung cancer. Ther Adv Med Oncol.

[5] Ding L, Getz G, Wheeler DA (2008). Somatic mutations affect key pathways in lung adenocarcinoma. Nature.

[6] Holt GE, Podack ER, Raez LE (2011). Immunotherapy as a strategy for the treatment of non-small-cell lung cancer. Therapy.

[7] Wang J, Zou ZH, Xia HL (2012). Strengths and weaknesses of immunotherapy for advanced non-small-cell lung cancer: a *meta*-analysis of 12 randomized controlled trials. PLoS One.

[b8] 8Lynch TJ, Bondarenko IN, Luft A, et al. Phase Ⅱ trial of ipilimumab (IPI) and paclitaxel/carboplatin (P/C) in first-line stage Ⅲb/Ⅳ non-small cell lung cancer (NSCLC). J Clin Oncol, 2010, 28(15s): abstr7531. http://www.asco.org/ASCOv2/Meetings/Abstracts?&vmview=abst_detail_view&confID=74&abstractID=53764

[9] Lynch TJ, Bondarenko I, Luft A (2012). Ipilimumab in combination with paclitaxel and carboplatin as first-line treatment in stage Ⅲb/Ⅳ non-small-cell lung cancer: results from a randomized, double-blind, multicenter Phase Ⅱ study. J Clin Oncol.

[10] Topalian SL, Hodi FS, Brahmer JR (2012). Safety, activity, and immune correlates of anti-PD-1 antibody in cancer. N Engl J Med.

[11] Butts C, Maksymiuk A, Goss G (2011). Updated survival analysis in patients with stage ⅢB or Ⅳ non-small-cell lung cancer receiving BLP25 liposome vaccine (L-BLP25): Phase ⅡB randomized, multicenter, open-label trial. J Cancer Res Clin Oncol.

[12] Thomas A, Hassan R (2012). Immunotherapies for non-small-cell lung cancer and mesothelioma. Lancet Oncol.

[13] Nemunaitis J, Nemunaitis M, Senzer N (2009). Phase Ⅱ trial of Belagenpumatucel-L, a TGF-beta2 antisense gene modified allogeneic tumor vaccine in advanced non small cell lung cancer (NSCLC) patients. Cancer Gene Ther.

[14] Bazhenova L, Carrier E, Shawler D (2012). Long-term survival in a Phase Ⅱ atudy of belagenpumatucel-L (TGF-β antisense modified tumor cell vaccine) in non-small cell lung cancer (NSCLC). Cancer Res.

[15] Vansteenkiste J, Zielinski M, Linder A (2007). Final results of a multi-center, double-blind, randomized, placebo-controlled Phase Ⅱ study to assess the efficacy of MAGE-A3 immunotherapeutic as adjuvant therapy in stage IB/Ⅱ non-small cell lung cancer (NSCLC). J Clin Oncol.

[16] Tyagi P, Mirakhur B (2009). MAGRIT: the largest-ever Phase Ⅲ lung cancer trial aims to establish a novel tumor-specific approach to therapy. Clin Lung Cancer.

[b17] 17Li R, Wang C, Liu L, et al. Autologous cytokine-induced killer cell immunotherapy in lung cancer: a Phase Ⅱ clinical study. Cancer Immunol Immunother, 2012 May 13. [Epub ahead of print]

[18] Xun YM, Xun DY, Zhang NZ (2010). Observation of chemotherapy combined with cytokine-induced killer cells and dendritic cells in patients with the advanced non-small cell lung cancer. Shi Yong Ai Zheng Za Zhi.

[19] Parikh PM, Vaid A, Advani SH (2011). Randomized, double-blind, placebo-controlled Phase Ⅱ study of single-agent oral talactoferrin in patients with locally advanced or metastatic non-small-cell lung cancer that progressed after chemotherapy. J Clin Oncol.

[20] Ren L, Zhou QH, Gou HF (2008). Study of the efficacy and survival time of the rmhTNF combined with chemotherapy in the treatment of non-small cell lung cancer. Hua Xi Yi Xue.

[21] Rouas-Freiss N, Moreau P, Menier C (2007). Expression of tolerogenic HLA-G molecules in cancer prevents antitumor responses. Semin Cancer Biol.

[22] Guo LH (2012). Immunological characteristics of tumor immune tolerance-tumor cells. Zhongguo Xi Bao Sheng Wu Xue Xue Bao.

[23] He W, Liu Q, Wang L (2007). TLR4 signaling promotes immune escape of human lung cancer cells by inducing immunosuppressive cytokines and apoptosis resistance. Mol Immunol.

[24] Kelly RJ, Gulley JL, Giaccone G (2010). Targeting the immune system in non-small-cell lung cancer: bridging the gap between promising concept and therapeutic reality. Clin Lung Cancer.

[25] Zheng X, Koropatnick J, Li M (2006). Reinstalling antitumor immunity by inhibiting tumor-derived immunosuppressive molecule IDO through RNA interference. J Immunol.

[26] Krieg AM (2006). Therapeutic potential of Toll-like receptor 9 activation. Nat Rev Drug Discov.

[27] Raynor J, Lages CS, Shehata H (2012). Homeostasis and function of regulatory T cells in aging. Curr Opin Immunol.

[28] Vignali DA, Collison LW, Workman CJ (2008). How regulatory T cells work. Nat Rev Immunol.

[29] Enioutina EY, Bareyan D, Daynes RA (2011). A role for immature myeloid cells in immune senescence. J Immunol.

[30] Wolchok JD, Hoos A, O'Day S (2009). Guidelines for the evaluation of immune therapy activity in solid tumors: immune-related response criteria. Clin Cancer Res.

